# A novel approach for Arabic business email classification based on deep learning machines

**DOI:** 10.7717/peerj-cs.1221

**Published:** 2023-01-25

**Authors:** Aladdin Masri, Muhannad Al-Jabi

**Affiliations:** Computer Engineering Department, An-Najah National University, Nablus, Palestine

**Keywords:** Machine learning, Email classification, Natural language processing, Arabic lexicon

## Abstract

During the last decades, the reliance on email communication, especially in business, has increased significantly. Companies receive a massive amount of emails daily, that include business inquiries, customers’ feedback, and other types of emails. This inspired many researchers to propose different algorithms to classify and redistribute the numerous emails according to their content. Nowadays, emails containing Arabic text, especially in the Arab world, have raised an increasing concern since they became widely used in official correspondence. Nevertheless, just a small amount of literature focuses on Arabic text classification. Therefore, this work addresses Arabic business emails classification based on natural language processing (NLP). A dataset of 63,257 emails was used and the emails were classified as: urgency, sentiment, and topic classification. The proposed models are based on machine learning techniques and a lexicon of words on which the emails are identified. The models are composed of different settings of convolutional neural networks (CNN). A separate model was built, trained, and tested for each category. The results were promising and gave an accuracy of about 92% and a loss of less than 8%. They also proved the correctness and robustness of this work.

## Introduction

Nowadays, email communication has a crucial role in almost all fields of our everyday life including businesses, health-care, education, society, and other fields. Therefore, there is a tremendous increase in the number of email messages exchanged. However, this increment is proportional to the number of unwanted emails that could be received. These unwanted emails could cause users to miss important ones. As stated in Dada et al. (2019), the user could spend momentous time reading unwanted emails such as spam, phishing, bulk messages, or even reading unimportant messages. In addition, the amount of spam emails reaches 77% of the global email traffic (Dada et al., 2019). Therefore, there is an increasing demand for email filtration and classification techniques in order to minimize the user time during reading the received messages.

Automatic email classification is an essential tool for email management. This tool automatically classifies emails into one or more predefined discrete categories. As stated in Mujtaba et al. (2017), one can benefit from a system that categorizes an incoming email into official, personal, phishing or normal, and spam or ham.

Moreover, the engines of abundant mail servers are using numerous authentication techniques to analyze the content of the email. To know whether to classify a new email as spam or not, its source is compared with a database of black and white lists (Bahgat et al., 2018). These lists can be optimized by users. An alternative technique is to filter emails by extracting features from the email body and using classification methods. This includes random forest (RF), support vector machine (SVM), naïve Bayes (NB), and neural networks (NN) (Bahgat et al., 2018; Saidani, Adi & Allili, 2020).

Machine learning showed also increasing importance in email classification and filtration. Deep learning natural language processing (NLP) has become very popular due to its capability to handle text, even if it is far from being grammatically correct. Machine learning techniques have been used to get modern results on NLP tasks like text classification, text ranking, question answering, relation classification, text summarization, machine translation, and others (Joshi, Goel & Joshi, 2020; Bianchi, Nozza & Hovy, 2021; Nguyen, Le & Nguyen, 2021).

The focus of this research is email classification of the Arabic language based on text classification. Text classification is the most extensively used NLP task. It is an essential component for intent detection in conversational systems. In literature, there have been few works focusing on text classification of the resource-constrained Arabic language (Saeed, Rady & Gharib, 2021; Al-Laith & Shahbaz, 2021) although Arabic is a morphologically rich and relatively free word order language. That is due to the unavailability of large training data in addition to the generalization of deep learning architectures to different languages (Touahri & Mazroui, 2021). Consequently, this work investigates the performance of deep learning models for Arabic text classification, because there has been a substantial rise in Arabic language digital content in recent years (R. Saeed, Rady & Gharib, 2021). For example, service providers, and e-commerce industries are now targeting local languages to improve their visibility. The originality of this work is that it deals with Arabic language classification using three different models, while most of the recent works focus only on sentiment analysis. It also aims to help in the selection of the right models and provide a suitable benchmark for further research in Arabic text classification tasks.

The article is structured as follows. The Literature Review section contains the related and previous works. The methodology and classification techniques are discussed in the Methodology section. In the Experiments and Results section, the detailed experiments are described and the results are presented. Finally, the Discussion and Conclusion section discusses the obtained results and contains the conclusion of this work.

## Literature Review

Due to the increasing number of email messages and the importance of communication worldwide, several aspects of email communication have the attention of many researchers. In Fang et al. (2020), the authors focused on forensics analysis by introducing a visualization model for email forensics of active relations aimed at mining social relationships and semantic patterns in emails. Other researchers focused on detecting attacks against systems. The work in Pitropakis et al. (2019) presented a taxonomy and survey of attacks against systems that use machine learning. Kumar, Chatterjee & García Díaz (2020) proposed a methodology for phishing detection consolidating feature extraction and mails classification using SVM. In addition, according to Sharma & Kumar (2017), machine learning methods are used to train the classifier on email messages to understand spam and non-spam messages, fraud detection, *etc*. Using machine learning methods in email content analysis facilitated the conformal prediction and information extraction, classification, and regression. For example, in Borg et al. (2021) and Borg, Boldt & Svensson (2019) the authors studied the large companies’ service improvement achieved by email classification.

On the other hand, email spam filtering and email classification attracted different researchers. In Dada et al. (2019), the work reviewed some of the popular machine-learning-based email spam filtering approaches. In Samira et al. (2020), the authors presented a hybrid technique for spam filtering relying on the Neural Network Model Paragraph Vector-Distributed Memory (PV-DM). Moreover, in Bahgat et al. (2018), the authors addressed an efficient email filtering approach based on semantic methods. The presented approach employs the WordNet ontology and applies different semantic-based methods and similarity measures for reducing the huge number of extracted textual features. Also, the work in Clark, Koprinska & Poon (2003) presented a neural network-based system for automated email filing into folders and anti-spam filtering. In Srinivasan et al. (2021), different email representation methods are proposed to transform emails into email word vectors, as a crucial step for machine learning algorithms.

The authors widely reviewed articles on email classification published between 2006 and 2016 by manipulating the methodological decision analysis (Mujtaba et al., 2017). In another work (Liu, Lee & Lee, 2020), the authors developed a framework for document-level multi-topic email data sentiment classification. They introduced an optional data augmentation process to enlarge the size of datasets with synthetically labeled data to avoid possible overfitting and underfitting during the training process. NLP attracted many researchers. As in Joshi, Goel & Joshi (2020), the authors surveyed deep learning architectures for tasks of text classification and they focused on Hindi text. Also, in Vijayan, Bindu & Parameswaran (2017), the authors conferred a detailed survey on the text classification process, and diversified algorithms used in this field.

In Suma & Kumara Swamy (2016), the authors used fuzzy logic methods for email clustering. To extract concept and feature, the keyword of the same feature goes into one cluster. If a new keyword is found and not matched with any existing cluster, then a new cluster is created for that. In Kulkarni & Shivananda (2019), the authors implemented deep learning for NLP based on different aspects: information retrieval using deep learning, text classification using CNN, recurrent neural network (RNN), and long short-term memory (LSTM), and predicting the next word/sequence of words using LSTM for emails. The main contribution of Cidon et al. (2019) was to divide the classification problem into two parts, one analyzing the email header, and the second applying NLP to detect phrases associated with suspicious links in the email body. In Peng, Harris & Sawa (2018), the authors presented an approach that uses NLP techniques to detect phishing attacks by analyzing text and detecting inappropriate statements. Prabha & Umarani Srikanth (2019) was a survey in which the authors focused on the different characteristics of the deep learning approaches used in various applications of sentiment analysis at the sentence level and aspect/target level. Moreover, in Alamoudi & Alghamdi (2021), the authors analyzed the content of a restaurant online reviews. The reviews are analyzed into two sentiment classifications, ternary classification (positive, negative, and neutral) and binary classification (positive and negative). They applied three different types of predictive models including machine learning, deep learning, and transfer learning models. The authors in Sueno, Gerardo & Medina (2020) used an improved NB algorithm to vectorize documents according to a distribution of probabilities reflecting the probable categories to which the document belongs.

Arabic is a semantically rich and relatively free word order language, and in recent years there has been considerable growth in Arabic language digital content. Naili, Chaibi & Ben Ghezala (2017) proposed a sentiment analysis framework that incorporates Arabic dependency-based rules and deep learning models. Also, Diwali et al. (2022) introduced a deep learning-based system for Arabic short answer scoring. The work aimed to provide a reliable system that can help teachers in the Arab world better use their time in other teaching activities that would increase the quality of learning in the region. Consequently, the contribution of this work is focusing on the classification of business emails using NLP for the Arabic language. Therefore, this work aims to propose and evaluate the performance of three deep learning models. Each model is used for a different Arabic text classification task. These models are email urgency, sentiment analysis, and topic classification.

## Methodology

This section discusses the methods used to achieve and classify the dataset as well as the methods used in the creation of each one of the used models. It also explains the training method of the models. The section has three main subsections. The first subsection is the dataset subsection, which discusses the dataset assemblage and classification approach. The second one discusses the creation and training of each model. The third one shows the application of the detection model in the three different cases.

### Data collection and preprocessing

**Figure 1:**
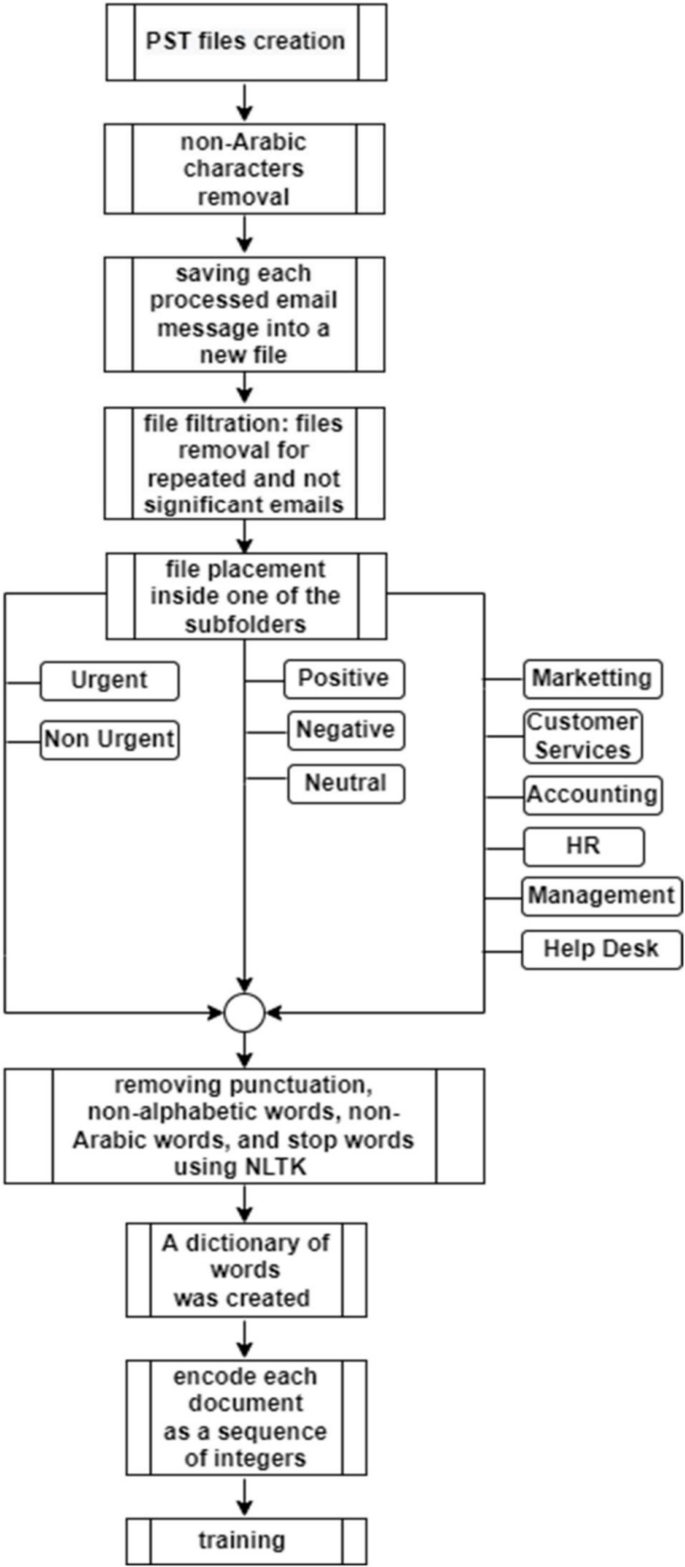
NLP-Dataset collection and filtration procedure.

This subsection discusses the dataset collection and classification technique, and the used transformation filters to convert the dataset into a usable Arabic-only dataset. Figure 1 shows the dataset collection and filtration procedure used in this research. The procedure is described as follows:

#### Data collection and classification

The dataset collection was performed through collaboration with Code Sky Technology (CST), which is a custom software development company. CST provided its domain emails from five different email accounts for this research. The email messages of each account were stored in a Personal Storage Table (PST) file. These email accounts are:

– The account info@codeskytech.com receives emails from anyone outside the company who wants to initiate a connection with the company.
– The CEO email account.
– The account for the head of the business development department.
– The account for the head of the marketing department.
– The account for the head of the sales department.

By using a Python program, each message inside the PST files is accessed. The python program reads every message in the file and removes any non-Arabic characters. It also removes any email that has no Arabic content. Every email is saved into a new file named according to a predefined counter. The number of emails at this stage was 87,458 emails.

In order to process and classify the produced emails, it was required first to remove all white spaces and any incoherent strings left by the deletion of non-Arabic characters. The next step of the email processing was filtering since many emails were repeated and others have no significance to the dataset (phishing or spam). The existence of a large number of such emails would shift the dataset toward wrong results. As a result of the filtration process, the final dataset included almost 63,257 finalized emails containing only the Arabic parts of the emails.

After processing, each email would be placed into at least one of the subfolders of each of the following three main folders:

1. Urgency: This folder has two subfolders: urgent and non-urgent.
2. Sentiment: This folder has three subfolders: positive, negative, and neutral.
3. Topic classification: This folder has six subfolders: marketing, human resources (HR), management, accounting, customer services, and help desk. This level of classification represents the main company departments.

Each email was classified according to its content and was stored in only one of these subfolders. These emails were used to train the three models: urgency model, sentiment analysis model, and topic classification model.

Next, a process of dataset preprocessing and presentation was needed after the classification process. Therefore, a word representation method needs to be selected. The word embedding method (Nael, ELmanyalawy & Sharaf, 2022) was selected because it provides a method for context preservation. Such that, Word embeddings are dense representations of the individual words in a text, considering the context and other surrounding words that individual word occurs with. It is the key breakthrough for the impressive performance of deep learning methods on challenging NLP problems (Brownlee, 2017). Moreover, this method uses neural networks to connect vector representations of words together, where the words that have the same meaning, have a similar representation. Also, since the implementation of text analysis especially with topic classification is a very context-dependent problem, word embedding is the most suitable data representation method for this work.

The use of the word embedding method implies that certain preprocessing techniques also had to be fit. Preprocessing is the final data preparation step which allows the models to have an accurate dictionary that will be used in their computations. The used preprocessing techniques include removing punctuation, non-alphabetic words, non-Arabic words, and stop words using the Natural Language Toolkit (NLTK) (Bird, 2006). A dictionary of words was also created using the words from the training set of each model.

In addition, it was required to encode each document as a sequence of integers, since the Keras embedding layer requires integer inputs. Each of these integers maps to a single token that has a real-valued vector representation. These vectors grow throughout the training process. With a more trained dataset, these vectors become more and more useful in creating more accurate representations of the words. The token creation was done using the Tokenizer class in the Keras API.

#### Testing, validating, and train splitting

To verify the correctness and accuracy of the proposed models, that dataset was tested in two different scenarios as follows:

1. In the first scenario, the dataset was separated manually (by the owner company) into several subsets according to each model’s categories: three subsets for the sentiment model, two subsets for the urgency model, and six subsets for the topic model. Then each subset of the dataset was tested by each corresponding category of each model.
2. The second scenario was to test the whole dataset by all the categories of each model.

#### Word emerging and preprocessing NLP

**Figure 2:**
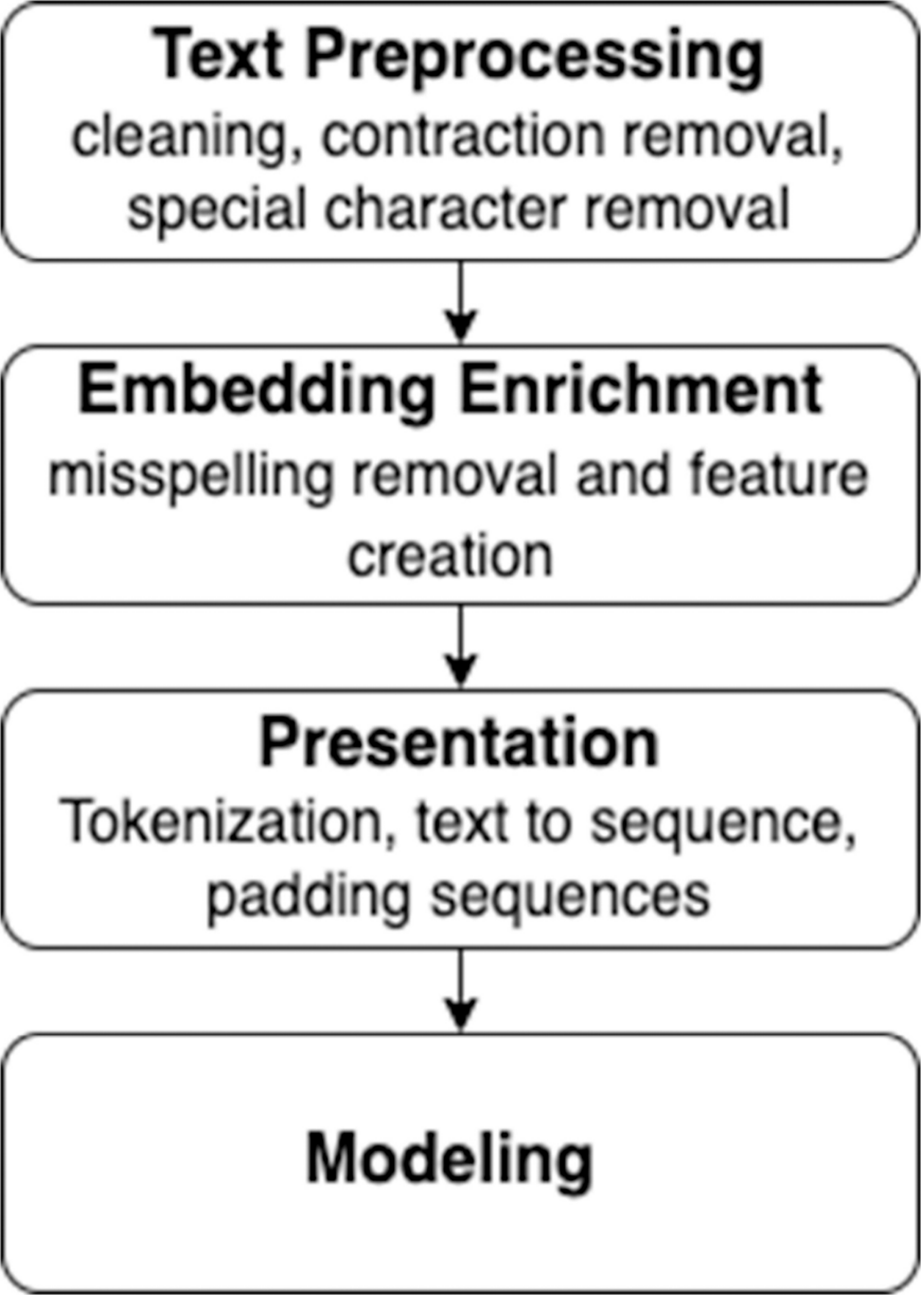
Text preprocessing infographic diagram.

Figure 2 describes the preprocessing of the data before going through all the steps to get a higher-level perspective about the whole process. Normally, the process starts by cleaning up the text data and performing some misspelling removal and feature creation to improve the textual input data quality. In addition, it is necessary to improve the quality of Word2Vec embeddings by removing Out-of-Vocabulary (OOV) words. The order of the first two steps has some flexibility, and it is generally permitted to go back and forth between these two steps. Next, some parameters have to be identified before training the models, for example, the size of the vocabulary, the number of unique words in the text, and the dimension of the embedded vectors. Therefore, a representation is needed for the text that could be fed into deep learning models. After that, creating and training models can be started. Finally, the last step is evaluating the models using appropriate metrics.

#### Model training

The fitting of the model was examined through 20 epochs with binary cross-entropy loss function and Keras Adam optimizer.

#### Performance metrics

To check the reliability and validity of the results, different metrics were used for both training and testing processes. F1 score, precision, and accuracy metrics were used. For instance:

– precision is calculated as the sum of true positives across all classes divided by the sum of true positives and false positives across all classes;
– accuracy is the total number of correct predictions divided by the total number of predictions made for a dataset;
– recall is calculated as the sum of true positives across all classes divided by the sum of true positives and false negatives across all classes.

F1-Measure provides a way to combine both precision and recall into a single measure that captures both properties, and can be calculated as in Eq. (1):

(1) \begin{eqnarray*}F1= \frac{2\ast \text{precision}\ast \text{recall}}{\text{precision}+\text{recall}} .\end{eqnarray*}

### Detection model

This subsection section describes the models’ creation, the algorithms used, and the training of the models. The models training was performed using a high-performance laptop, a DELL Inspiron 15,7000 Gaming Intel(R) Core(TM) i7-7700HQ CPU @ 2.80 GHz CPU with 16 GB RAM. The anaconda Jupyter Notebook platform was used, with Python 3.8 programming language. In addition, to predict the most suitable values of the proposed models’ parameters, the model was evaluated and examined 10 times for different values.

As for the models’ creation, a result-driven model modification method was used, starting with a basic model, influenced by the models found in Brownlee (2017) such as character-based or word-based NLP. Then, the models were adjusted based on the results of each iteration to achieve the best possible results with the highest possible accuracy. This optimization includes the number of filters used, kernel size, activation function, and the number of layers. This operation was executively challenging since the Arabic language has significant differences when compared to English. Since the Arabic language has special characteristics, additional difficulties were added to the dataset, such that all Arabic characters that appeared as invariants were rendered into a single common character. For example: ‘T marbotah’ ( 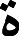) replaced by ( 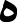), ( 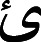) replaced by ( 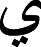), and hamza ( 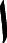, 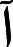, 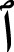, 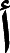, 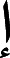 ) replaced by ( 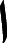) (Aljuhani, Alyoubi & Alotaibi, 2022). In addition, based on diacritics and dialect, the word 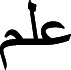 can have different meanings: taught, knowledge, flag, *etc.* This is a common phenomenon in Arabic that makes the language rich and complex (Nael, ELmanyalawy & Sharaf, 2022).

Therefore, a change was needed in the proposed models. The Keras embedding layer was used in all of the models, as explained in the previous section. One important point is the use of a Length L property, which represents the number of words the model receives.

**Figure 3:**
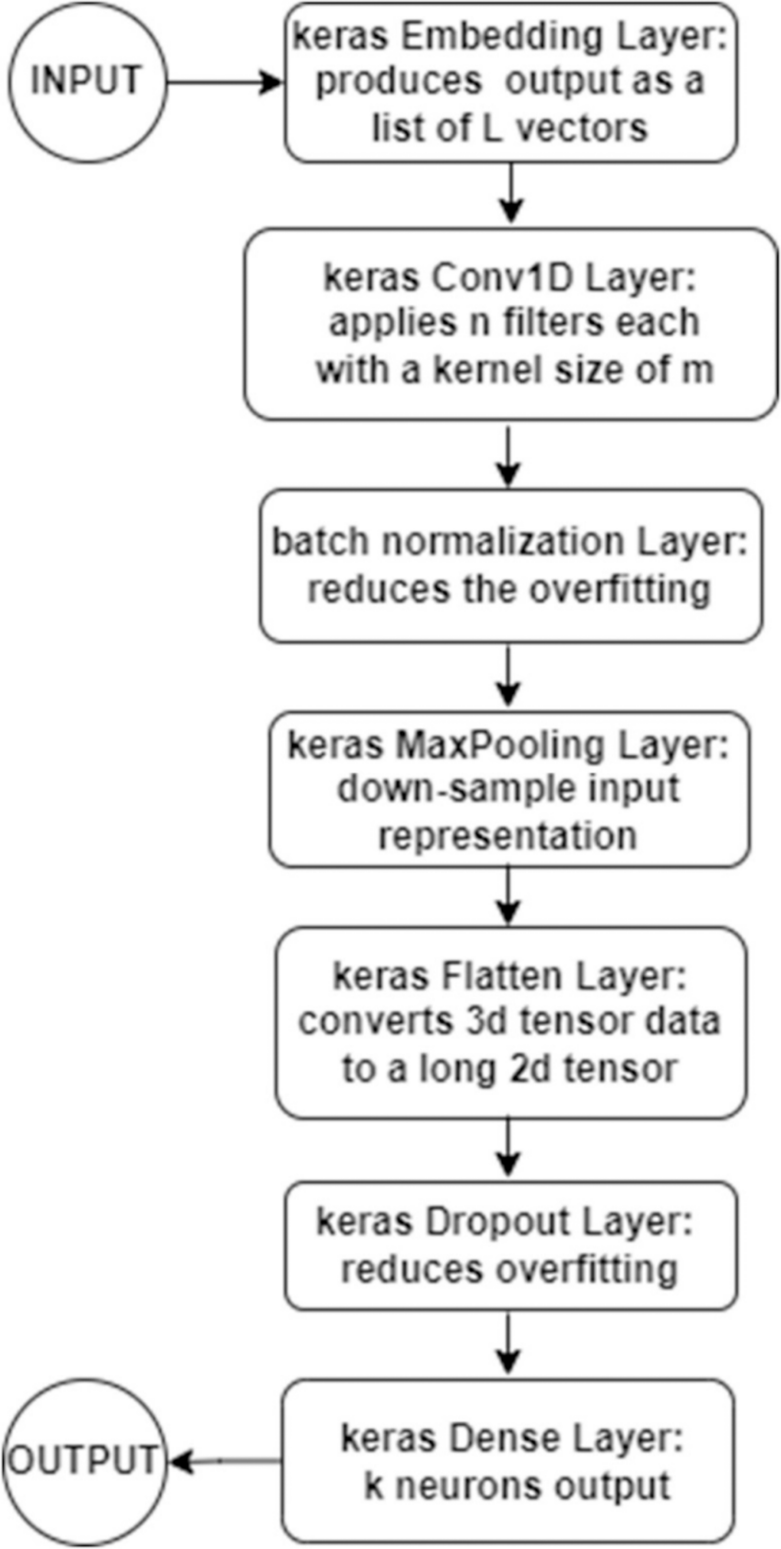
Detection model flowchart.

Initially, a detection model was created as shown in Fig. 3. The Keras embedding layer was used as an input layer that takes a document with L words (maximum size of 500 words) and produces the output as a list of L vectors. Each vector represents a distinct word in the document and has a length of L. The output was then fed to a 1D convolution Keras layer CNN that applies m filters with a kernel size of 8. Then a batch normalization layer was added to reduce the overfitting. Followed by Keras max pooling layer as a pooling layer, it was used in order to down-sample the input representation, reduce its dimensionality, enhance the feature-extracting process and reduce the computational cost. The next layer in the implementation was the Keras flatten layer, which was used to convert the 3d tensor data to a long 2d tensor. This 2d tensor data was then fed to the next layer which is a Dropout layer to reduce the overfitting. The output was finally fed to a Keras dense layer which provides k neurons as the final output shown in Eq. (2):

(2) \begin{eqnarray*}\text{Output}=\text{Activation}(\text{dot} \left( \text{input.kernal} \right) +\text{bias})\end{eqnarray*}

where the activation function is sigmoid (logistic) activation function.

### Applying detection model

The model in Fig. 2 was applied for urgency, sentiment, and topic detection cases. The following tunings were made for each case:

(a) For urgency detection, the Keras embedding provided a list of 512 vectors, while the number of the 1D convolutional Keras layer filters (m) was 64. In the dense layer, two dense layers were used, one as the last hidden layer with 10 neurons and the second as the output layer with 1 neuron, which represents the final output for the urgency of the provided text.
(b) The model tuning for sentiment analysis was made by altering the final output layer to have three neurons as output, one for positive sentiment, one for neutral, and one for negative sentiment. In addition, the output vector length of the Keras embedding layer was reduced to 256, and the number of filters of the 1D convolutional Keras layer (m) was reduced to 32.
(c) The model tuning for topic classification was performed by adjusting the Keras embedding layer vectors to 256, the 1D convolutional Keras layer filters (m) to 64 and the first dense layer to have 256 neuron outputs, and the second dense layer to have six neurons, each representing one of the six topics to which the classification refers.

## Experiments and Results

In this section, the results of this research will be discussed and evaluated. The experimental dataset consists of 63,257 emails used in training and testing the proposed models. The experiments were conducted with three different classification models: sentiment, urgency, and topic. The experiments measure the accuracy based on the sample taken from the dataset for training and testing each model. To perform the experiments, a subset of the data (80%) was used for training and the rest (20%) for testing. The results were promising and affirmed the correctness of the work in this research.

### Sentiment model analysis

To test the data for sentiment analysis, the dataset was classified into three categories: positive, negative, and neutral. Table 1 shows the results and the used metrics for each category. It also shows the results for the overall dataset.

**Figure 4:**
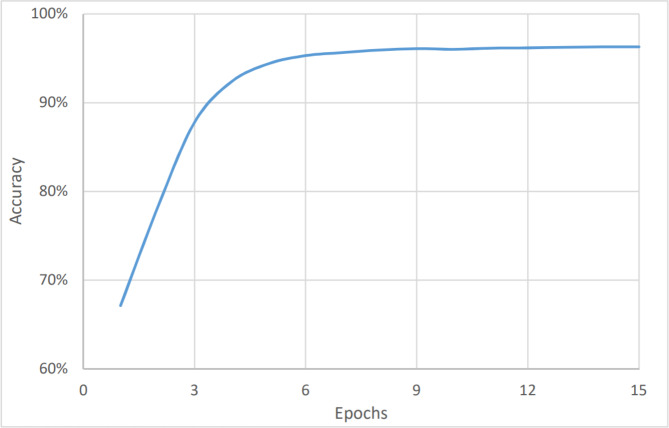
Sentiment analysis, accuracy *vs* epochs.

**Figure 5:**
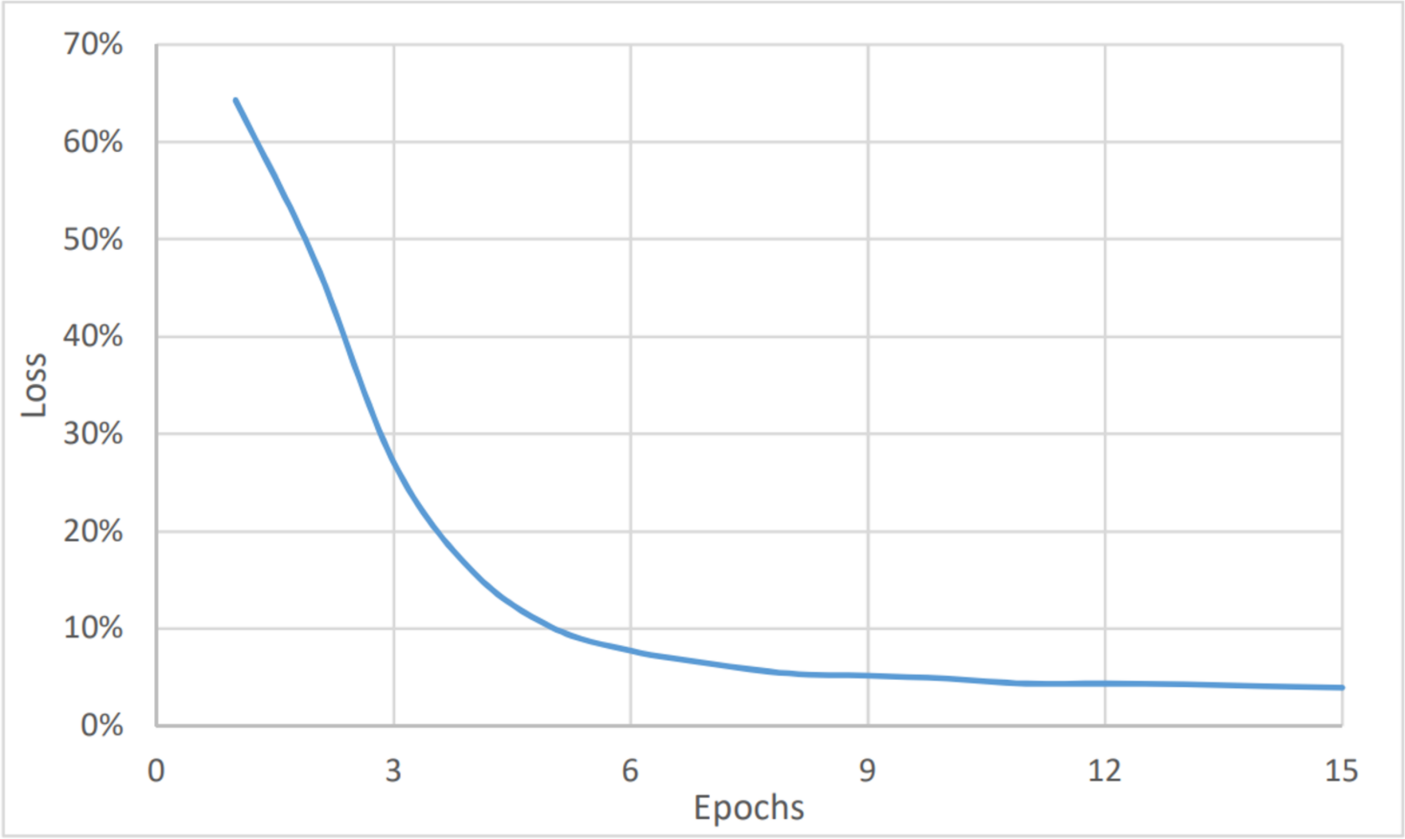
Sentiment analysis, loss *vs* epochs.

On the other hand, Figs. 4 and 5 show the relationship between the accuracy and loss *vs* the number of epochs respectively. The figure shows that the accuracy is increasing gradually during the first few epochs or training iteration before it becomes stable at about 96.1% (after nine epochs), while the loss is decreasing continuously during the initial number of epochs before it becomes stable at 3.9%. The average timing of each epoch was 1480s.

### Urgency model analysis

**Figure 6:**
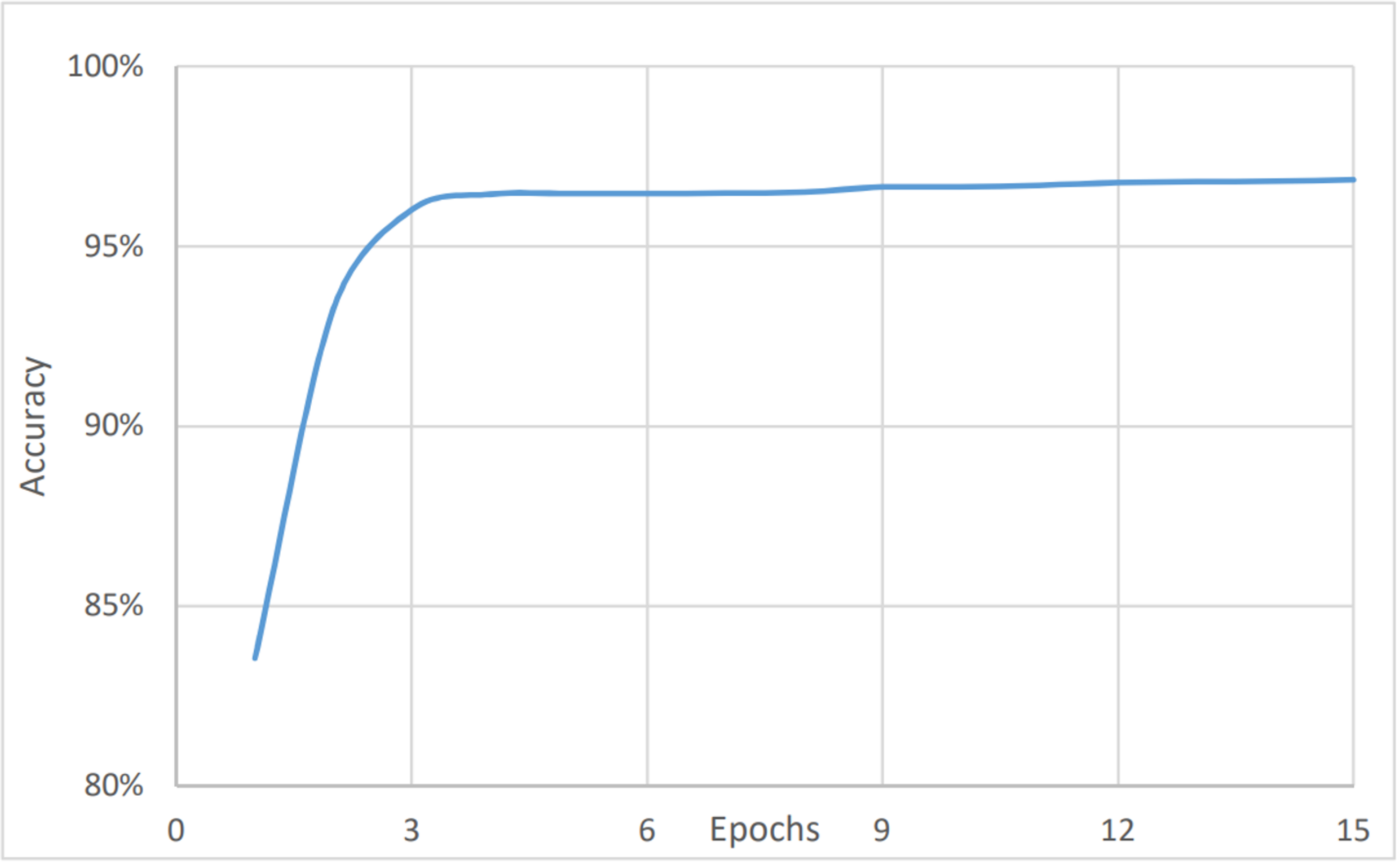
Urgency analysis, accuracy *vs* epochs.

**Figure 7:**
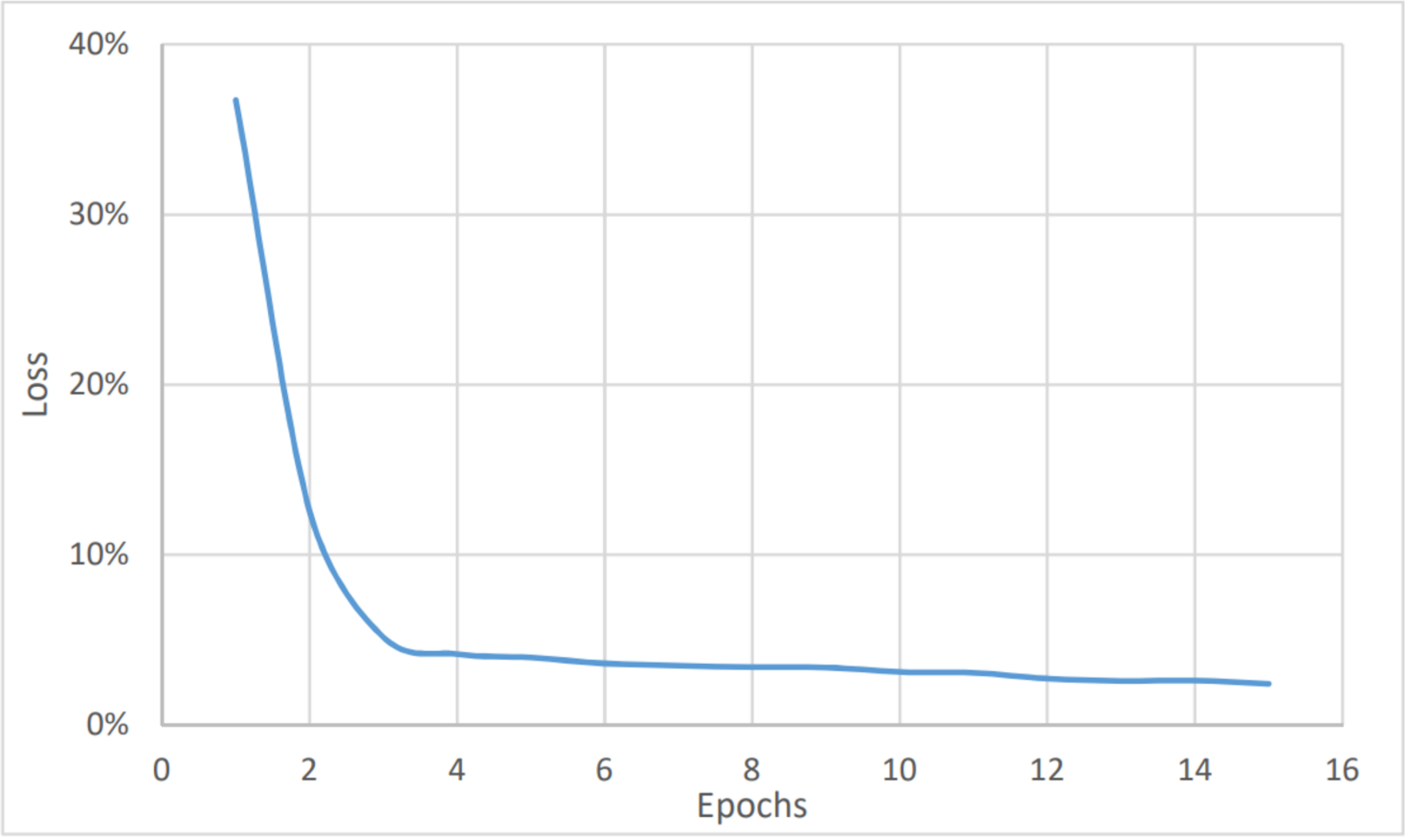
Urgency analysis, loss *vs* epochs.

To perform the urgency analysis, the dataset was classified into two categories: urgent, and non-urgent data. Table 2 shows that the precision, accuracy, and F1 score were better since there were two categories only. This was approved by the convergence of the accuracy value to 97% (after eight epochs) as shown in Fig. 6. while the loss value is converging to 2.4% as shown in Fig. 7. The average timing of each epoch was 1440s.

### Topic model analysis

**Figure 8:**
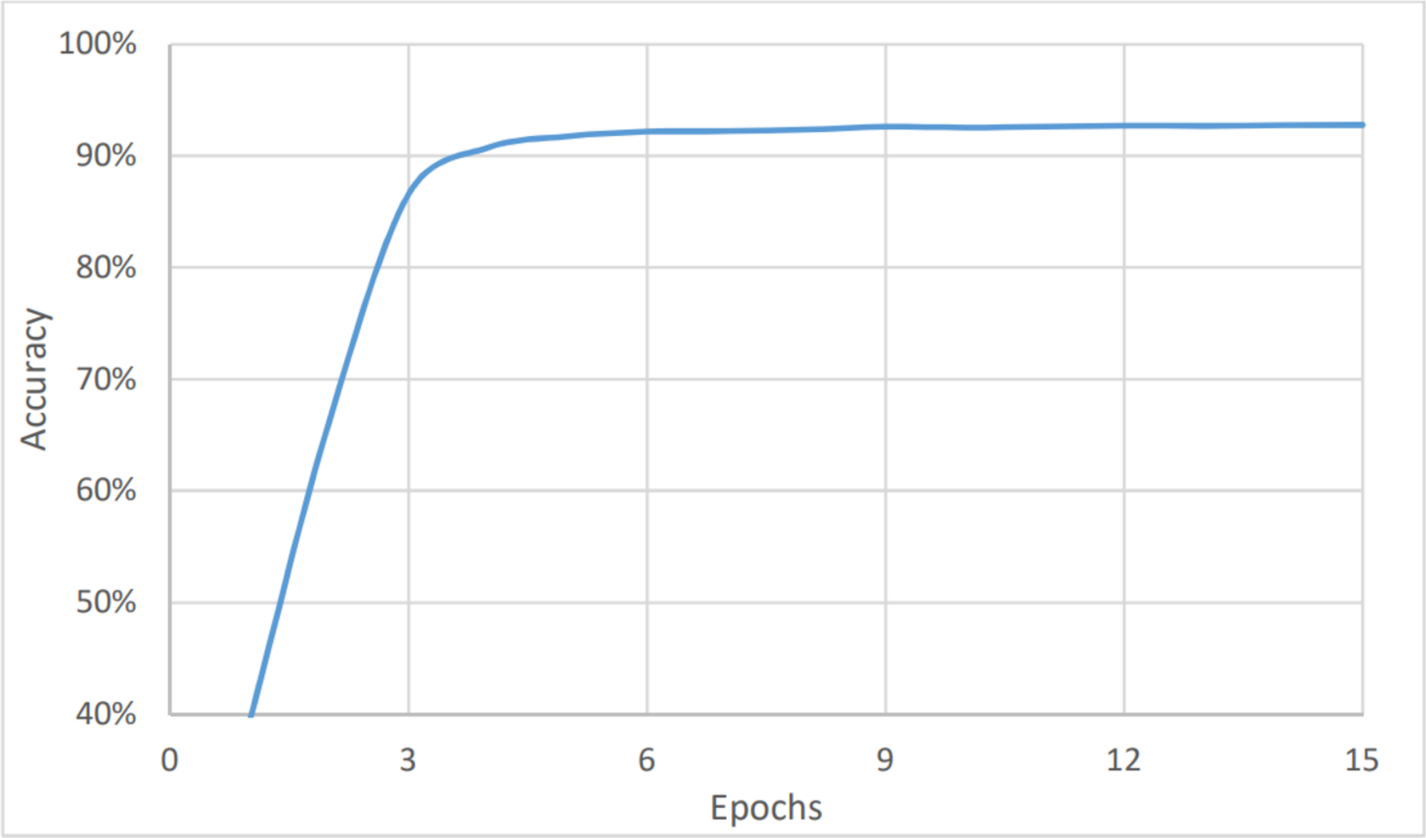
Topic analysis, accuracy *vs* epochs.

**Figure 9:**
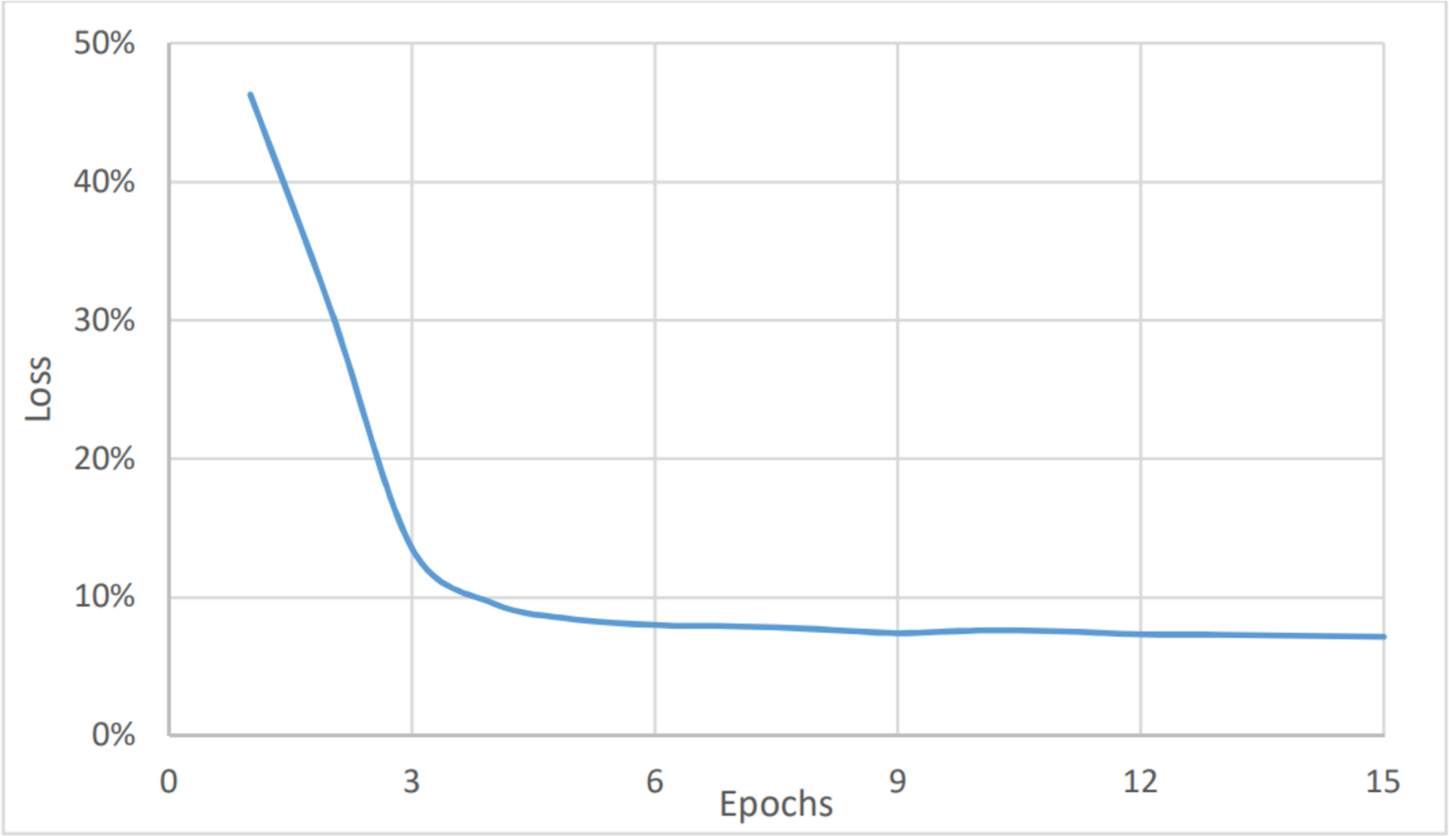
Topic analysis, loss *vs* epochs.

The last measure was for the topic analysis. As with the two aforementioned models, the dataset was classified into six categories: marketing, customer services, accounting, HR, management, and help desk. Here, the metrics values were the lowest for the complete dataset, as shown in Fig. 8, and in Fig. 9, and Table 3. This is because the number of categories has increased. Figure 7 shows that the value of accuracy after a number of epochs has been stained at a value of about 92% (after nine epochs), while Fig. 8 shows a higher loss. The loss percentage after a number of epochs reached a value of 7.2%. The average timing of each epoch was 1560s.

**Table 1:** Sentiment model analysis for different categories and metric values.

	Category	Dataset size	Detected emails	Precision	F1 score	Accuracy
Scenario 1	Positive	40,548	38,845	95.5%	96.1%	95.8%
	Negative	9,729	8,415	87.1%	87.6%	86.5%
	Neutral	12,980	11,721	90.4%	91.9%	90.3%
Scenario 2	Overall data	63,257	56,805	89.9%	90.4%	89.8%

## Discussion and Conclusion

**Figure 10:**
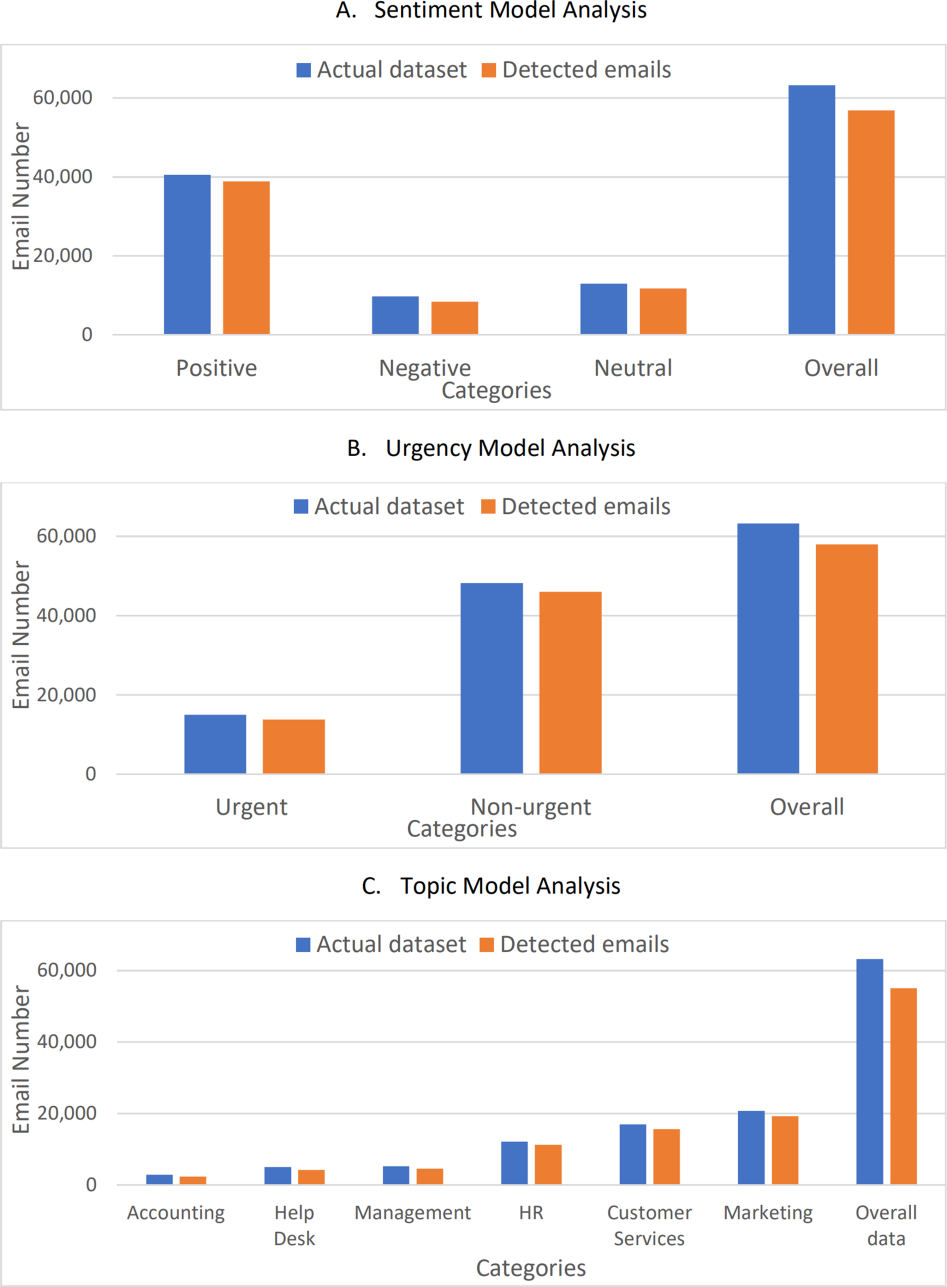
Comparison between the actual datasets and the detected emails. (A) Sentiment model analysis. (B) Urgency model analysis. (C) Topic model analysis.

Figure 10 shows a comparison between the actual datasets and the detected emails. The figure shows clearly that the values are very close with an average accuracy of about 92% with a loss of less than 8%. To verify the correctness and accuracy of the results, the results were compared to the values found in the comparative study for Arabic NLP Syntactic Tasks (Abushaala & Elsheh, 2022) and a study of Arabic sentiment analysis using Naïve Bayes and CNN-LSTM (Suleiman, Odeh & Al-Sayyed, 2022). The work in Suleiman, Odeh & Al-Sayyed (2022) showed also a complete comparison of the recently proposed sentiment analysis approaches. The work in Abushaala & Elsheh (2022) concerns comparing different deep learning techniques based on data for Arabic NLP lexical and syntactic tasks. Table 4 shows the F1-score of the different models tested, while the work in Suleiman, Odeh & Al-Sayyed (2022) summarized most of the recently proposed Arabic sentiment analysis classifiers. Table 5 shows the accuracy values for the recently proposed sentiment analysis approaches.

Comparing the results obtained from this work with the results from Abushaala & Elsheh (2022) and Suleiman, Odeh & Al-Sayyed (2022) gives:

**Table 2:** Urgency model analysis for different categories and metric values.

	Category	Dataset size	Detected emails	Precision	F1 score	Accuracy
Scenario 1	Urgent	15,033	13,815	92.0%	92.6%	91.9%
	Non-urgent	48,224	46,054	95.5%	96.5%	95.5%
Scenario 2	Overall data	63,257	58,007	91.2%	92.1%	91.7%

(1) The work in Abushaala & Elsheh (2022) and Suleiman, Odeh & Al-Sayyed (2022) focuses on the Arabic sentiment analysis while the work presented in this article presents the analysis of Arabic language with three different models (topic sentiment and urgency). This confirms the novelty and originality of this work.
(2) Both F1-score and accuracy shown in Abushaala & Elsheh (2022) and Suleiman, Odeh & Al-Sayyed (2022) were equal to or below the results in this work. Hence, the results of this work were more accurate. Such accuracy and loss values are used to confirm the reliability and validity of the proposed models.

With the fast spread of the Internet, emails facilitated data and information exchange for both personal and business-related aspects. In this research, a novel approach for classifying Arabic emails in different data sets is presented. The work aimed to introduce a model that can facilitate classifying received emails and direct them to the correct person in each department. The model helps in enhancing the performance of email classification based on the deep learning technique. The work dealt with different types of classification: sentiment, urgency, and topic analysis. The model was tested on a dataset of 63,257 emails and the results reflected the correctness of the presented approach with about 92% of accuracy. The presented model can be customized according to the needs of use and can handle large datasets. The key of this work is to deal with Arabic emails classification with new aspects that few researchers have focused on. The main challenge of such work is acquiring the dataset, since it is either private or has a small size (Himdi, 2022; Cumaoğlu, 2022).

**Table 3:** Topic model analysis for different categories and metric values.

	Category	Dataset size	Detected emails	Precision	F1 score	Accuracy
Scenario 1	Accounting	2,939	2,383	81.3%	82.7%	81.1%
	Help Desk	5,053	4,270	84.1%	85.3%	84.5%
	Management	5,285	4,608	88.1%	88.5%	87.2%
	HR	12,201	11,298	92.6%	93.1%	92.6%
	Customer services	17,003	15,627	91.8%	92.7%	91.9%
	Marketing	20,776	19,343	93.3%	94.5%	93.1%
Scenario 2	Overall data	63,257	55,097	87.2%	88.9%	87.1%

In the future, this research can be boosted by using other effective classifiers, and other machine learning techniques such as RNN, LSTM and GRU with a larger and enhanced dataset (for more categories classification). The study can be also expanded to include semi-supervised deep learning approaches such as consistency regularization or proxy-label methods. Moreover, algorithms can be built to adjust the parameters automatically.

**Table 4:** F1-score of part-of-speech models tested by test datasets for 40 epochs (Abushaala & Elsheh, 2022).

Model	F1 score
NLP (the proposed model)	90.4%
LSTM	80.20%
BLSTM	80.40%
LSTM-CRF	79.70%
BLSTM-CRF	81.10%

**Table 5:** Recently proposed sentiment analysis approaches (Suleiman, Odeh & Al-Sayyed, 2022).

Year	Model	Dataset	Polarity	Accuracy
2023	NLP (the proposed model)	Arabic business email	Positive, negative or neutral	96.1%
2021 Chouikhi, Chniter & Jarray (2021)	Arabic BERT tokenizer	ASTD, HARD, LABR, AJGT, ArSenTD-Lev	(1) (ASTD) positive, negative or neutral (2) (HARD) positive or negative (3) (AJGT) positive or negative	96.1%
2022 Omara, Mosa & Ismail (2022)	deep LSTM, GRU, and CNN	Merges thirteen sets from free accessible sentiment analysis corpora	Positive, negative or neutral	95.1%
2020 ElJundi et al. (2019)	CNN+LSTM +SVM	Multi-domain sentiment corpus	Positive or negative	90.8%
2020 Kwaik et al. (2020)	Distant supervision approaches	ATSAD, LABR, ASTD, Shami-Senti	Positive or negative	86.0%.

##  Supplemental Information

10.7717/peerj-cs.1221/supp-1 Supplemental Information 1 Sentiment Training

10.7717/peerj-cs.1221/supp-2 Supplemental Information 2 Topic Training

10.7717/peerj-cs.1221/supp-3 Supplemental Information 3 Urgency Training

10.7717/peerj-cs.1221/supp-4 Supplemental Information 4 Vocabulary

10.7717/peerj-cs.1221/supp-5 Supplemental Information 5 Topic

10.7717/peerj-cs.1221/supp-6 Supplemental Information 6 Urgency

10.7717/peerj-cs.1221/supp-7 Supplemental Information 7 Sentiment

## Additional Information and Declarations

### Data Availability

The following information was supplied regarding data availability: The raw measurements are available in the Supplementary Files.
